# Pulmonary artery pseudoaneurysm causing massive hemoptysis in hyperimmunoglobulin E syndrome: a case report

**DOI:** 10.1186/s12890-019-0797-7

**Published:** 2019-02-08

**Authors:** Aaron Hakim, Isabel S. Bazan, Mamadou L. Sanogo, Edward P. Manning, Jeffrey S. Pollak, Geoffrey L. Chupp

**Affiliations:** 1 Yale University School of Medicine, New Haven, CT USA; 2 Section of Pulmonary, Critical Care, and Sleep Medicine, Department of Internal Medicine, Yale University School of Medicine, New Haven, CT USA; 3 Section of Vascular and Interventional Radiology, Department of Radiology and Biomedical Imaging, Yale University School of Medicine, New Haven, CT USA

**Keywords:** Massive hemoptysis, Pulmonary artery pseudoaneurysm, Hyperimmunoglobulin E syndrome

## Abstract

**Background:**

Hyperimmunoglobulin E syndrome (HIES) is a rare primary immunodeficiency disorder defined by high serum immunoglobulin E titers that is associated with recurrent respiratory infections, formation of pneumoatoceles, recurrent skin abscesses, and characteristic dental and skeletal abnormalities.

**Case presentation:**

We report a case of a 56-year-old male with a history of HIES, cavitary mycetomas, and allergic bronchopulmonary aspergillosis who presented with recurrent massive hemoptysis. Bronchial artery angiography and bronchoscopy failed to identify active hemorrhage, and two embolizations of the bronchial artery did not resolve the bleeding. Subsequently, selective pulmonary artery angiography was conducted that demonstrated a subsegmental pulmonary artery branch pseudoaneurysm with extravasation into an adjacent lung cavity. This was treated successfully with transcatheter embolization.

**Conclusions:**

To our knowledge, this is the first case reported of pulmonary artery pseudoaneurysm in HIES in the medical literature. Pulmonary artery pseudoaneurysm should be considered in the differential diagnosis in patients with HIES and massive hemoptysis.

## Background

Autosomal dominant hyperimmunoglobulin E syndrome (HIES), due to STAT3 mutations, is a rare primary immunodeficiency disorder characterized by eczema, elevated serum IgE, recurrent infections, and connective tissue and skeletal findings [1]. HIES patients develop pneumatoceles, likely due to aggressive and dysregulated inflammation [2]. Massive hemoptysis, defined as > 300 cc of blood expectorated within 24 h, is a major cause of mortality and morbidity in patients with HIES [3]. Two case reports of bronchial artery pseudoaneurysm causing massive hemoptysis have been described in HIES patients, both of which were managed with bronchial artery embolization [4, 5]. As of the date of publication, this is the first case reported in the literature of pulmonary artery pseudoaneurysm causing massive hemoptysis in a patient with HIES.

## Case presentation

**Fig. 1:**
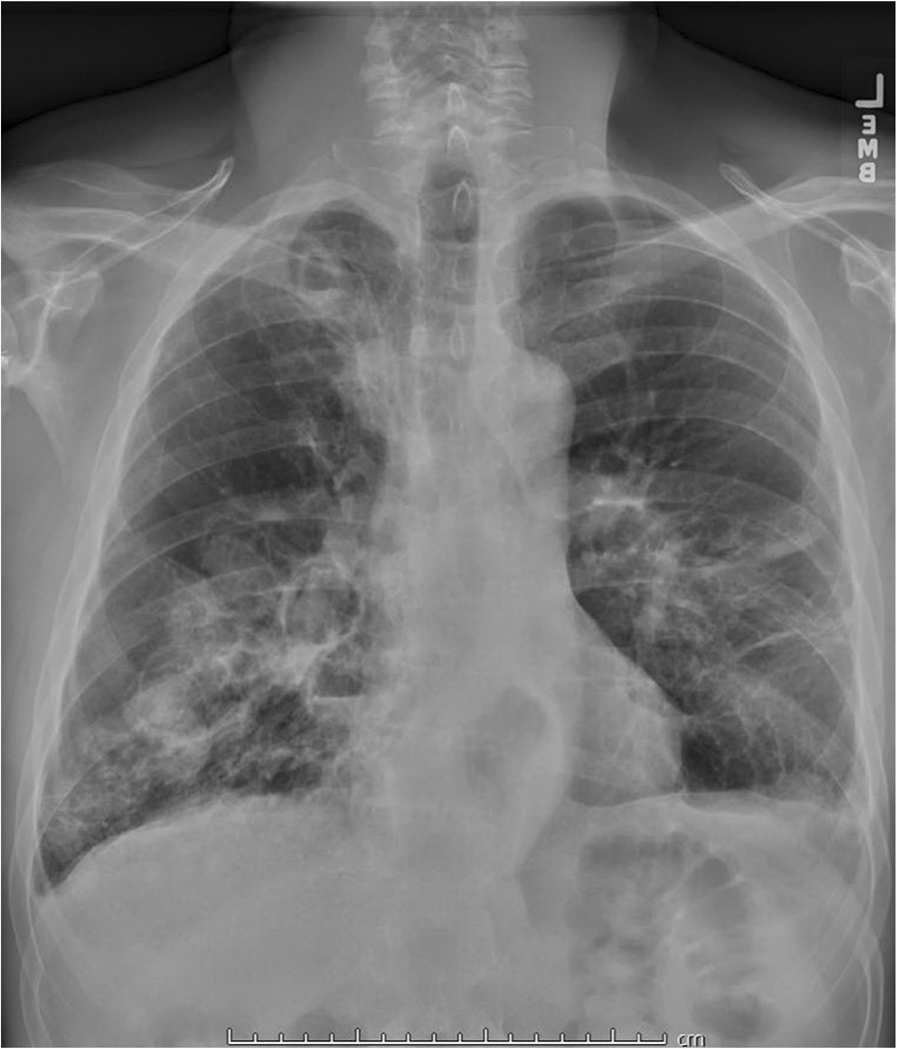
Chest x-ray (PA) revealing bilateral cystic bronchiectasis, scattered areas of scarring, and round lesions in the cavities compatible with mycetoma

**Fig. 2:**
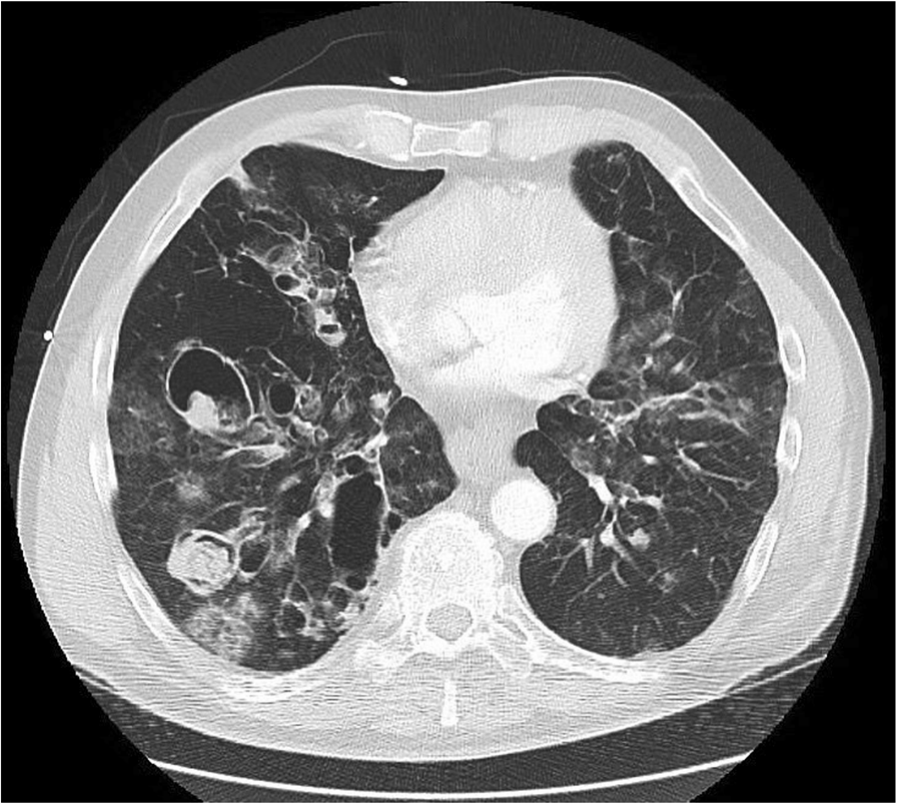
Computed tomography revealing multiple pneumatoceles and cavities with Aspergilloma, predominately in the bilateral lower lobes, right greater than left. The largest one is 6.4 cm in size. A right lower lobe pneumatocele was ultimately found to be associated with the pulmonary artery pseudoaneurysm

A 56-year-old man was admitted to the hospital for the management of recurrent massive hemoptysis. The patient had a history of recurrent pneumonia, including an episode complicated by empyema requiring a left lower lobe thoracotomy, and recurrent sinus infections requiring surgery. In addition, he had a long-standing history of pneumatoceles, cystic bronchiectasis (Fig. 1), multiple Aspergillomas on CT (Fig. 2), and allergic bronchopulmonary aspergillosis (ABPA) with sputum cultures positive for Aspergillus. Aspergillomas had been treated in the past with intravenous (IV) amphotericin B and oral itraconazole, right upper lobe lung wedge resection, IV capsofungin therapy, and most recently, participation in the National Institutes of Health anti-microbial treatment protocol for HIES with trimethoprim sulfamethoxazole, posaconazole, and prednisone [6] for the past 9 years. Other medical history included a diverticular abscess, a Mallory-Weiss tear, osteopenia, scoliosis, and gastroesophageal reflux disease. His family history was unremarkable. Prior investigations revealed eosinophilia, elevated serum immunoglobulin (Ig) E levels of 31,850 kU/l (normal range 0 to 115 kU/l), and normal IgA, IgG and IgM levels. The diagnosis of HIES was confirmed by the detection of a sporadic STAT3 mutation.

The patient first presented with persistent blood-tinged sputum and hematemesis 8 months prior to admission. At that time, esophagogastroduodenoscopy was done to rule out upper gastrointestinal bleeding. A chest CT with contrast for bronchial circulation showed a RLL basilar infiltrate consistent with blood, but no extravasation of contrast into the lung parenchyma. Arteriography of the right bronchial artery showed mildly abnormal vascularity without frank arterial extravasation or secondary angiographic signs of hemoptysis. The right bronchial and right 8th posterior intercostal arteries were embolized using 500–700 μm polyvinyl alcohol particles. The patient’s hemoptysis ceased and he was discharged home after 24 h.

The patient had no further hemoptysis for approximately 7 months and then developed sub-acute hemoptysis of approximately 10-20 cc per day for 2 weeks. After treatment with a course of oral antibiotics, he was admitted for evaluation. Chest CT without contrast showed no new abnormalities. Bronchoscopy did not demonstrate any active bleeding or an endobronchial source of bleeding. The hemoptysis improved and the patient was discharged home, but was re-admitted 23 days later after several episodes of hemoptysis > 200 cc. Laryngocospy was done to rule out sino-pharyngeal sources of bleeding. Percutaneous instillation of Amphotericin B paste into a cavitary mycetoma was considered [7, 8], but it was not clear which cavity was bleeding. Bronchial arteriography was repeated. Mild abnormal vascularity in the right lung was supplied by a slightly enlarged, tortuous right bronchial artery, but no active hemorrhage was observed. Notably, more intense bronchial vascularity is typically seen in the setting of Aspergillomas [9, 10]. Repeat embolization of the right bronchial artery and the distal right 6th intercostal artery was achieved using 250–355 μm polyvinyl alcohol particles. The hemoptysis stabilized and the patient was discharged after 4 days.

**Fig. 3:**
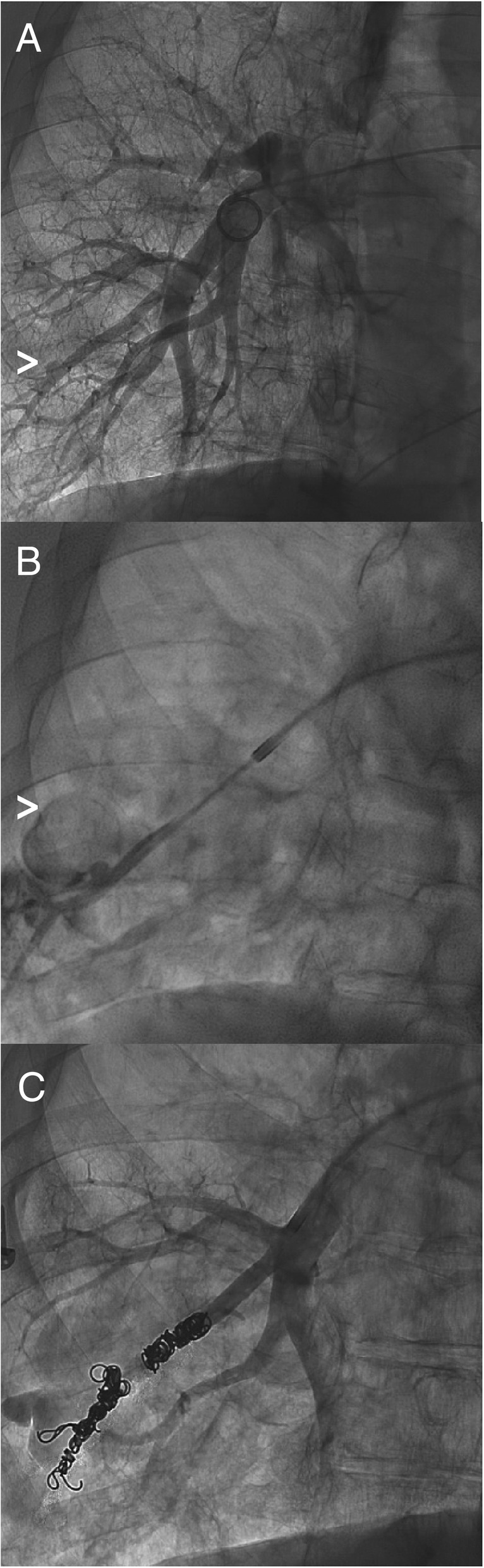
**a** Pulmonary artery angiography, right anterior oblique view 33^o^, showing small outpouching (arrowhead) from a subsegmental branch in the lateral right lower lobe. **b** The subsegmental branch was selected, and contrast actively extravasated into an adjacent airspace (arrowhead), at which point the patient also developed 250 cc of frank hemoptysis during the procedure. **c** Embolization was achieved using Nester coils (Cook, Bloomington, IN) and Amplatzer Vascular Plug II (St. Jude Medical, St. Paul, MN), with no further filling of the pseudoaneurysm. The patient’s hemoptysis had stopped by this time

The patient experienced another episode of massive hemoptysis the following week and underwent interrogation of the pulmonary arteries via angiography. This revealed a small outpouching from a subsegmental pulmonary artery branch in the lateral segment of the right lower lobe (Fig. 3a). Contrast injection within the subsegmental branch confirmed the presence of the small pseudoaneurysm, with contrast actively extravasating into an adjacent cavity in the lung (Fig. 3b). The patient developed gross hemoptysis at this time, and was suctioned for 250 cc of blood. Embolization was achieved using seven 0.035 in. gauge Nester coils (Cook, Bloomington, IN) distal, across, and proximal to the orifice of the pseudoaneurysm, along with one 6 mm Amplatzer Vascular Plug II (St. Jude Medical, St. Paul, MN) in between the coils. After this branch was occluded, no further filling of the pseudoaneurysm or extravasation was seen (Fig. 3c), and the patient’s hemoptysis resolved. The patient had no further hemoptysis and was discharged home, with no further hemoptysis as of this writing.

## Discussion and conclusions

We present the first reported case of pulmonary artery pseudoaneurysm causing massive hemoptysis in a patient with Hyperimmunoglobulin E syndrome and chronic cavitary Aspergillosis. Massive hemoptysis as a complication of Aspergilloma is uncommon but can be life-threatening, with an estimated mortality rate of 38% [11]. Although most patients with hemoptysis bleed from the bronchial circulation [12], our case highlights that the pulmonary arterial circulation should be evaluated if the bronchial angiogram does not reveal a source of bleeding. Furthermore, if the bronchial arteriogram is unrevealing in a patient with hemoptysis and chronic cavitary Aspergillosis, the pulmonary arterial circulation should potentially be studied in the same sitting to eliminate a delay in diagnosis, decrease the number of angiographic procedures, and avoid putting the patient at risk of a fatal episode of hemoptysis.

There are several potential mechanisms of pseudoaneurysm formation in HIES. Infections are common in HIES and may play a central role in the formation of vascular anomalies. The most commonly reported cases of aneurysm formation in HIES are of the coronary, carotid, and intracranial arteries [13]. Interestingly, vascular anomalies may appear in HIES without clear histopathologic demonstration that the inflammatory changes are related to infection [13]. It has been postulated that cytotoxic substances released from perivascular eosinophils may result in direct medial destruction, predisposing to aneurysmal formation, or alternatively that mutations in the STAT3 protein, which regulates vascular endothelial growth factor (VEGF), could contribute to vascular abnormalities. Additional studies will be required to understand the relative contributions of infection, eosinophilia and genetics to the pathogenesis of vascular anomalies in HIES.

The management of hemoptysis in the immunocompromised patient can be clinically challenging, and despite being a common complication in HIES, no guidelines exist for the treatment of hemoptysis in HIES. Lung parenchymal surgery in HIES has a complication rate exceeding 50%, often resulting in bronchopleural fistulae necessitating prolonged antibiotics, prolonged thoracostomy tube drainage, and re-operations [14]. Percutaneous intracavitary instillation of Amphotericin B is an investigational approach for the treatment of massive hemoptysis from pulmonary Aspergilloma [7, 8]; however, efficacy has not been demonstrated by randomized trials, and intracavitary instillation may be technically challenging in patients with multiple mycetomas where the culprit lesion is often unclear and/or unfavorably located within the lung. Transcatheter embolization techniques with coils or plugs have proven effective in treating massive hemoptysis, and the use of covered stents within adult pulmonary arteries has also been described [15]. These non-surgical approaches have demonstrated short-term non-recurrence rates of 73–98% for bronchial artery embolization and long-term recurrence of hemoptysis in 10–52% of patients [16, 17]. An exception is bronchial artery embolization for Aspergilloma that has especially high rate of hemoptysis recurrence and mortality [18].

In summary, this case represents a novel and rare complication of HIES and illustrates the clinical challenges in diagnosing and managing persistent recurrent hemoptysis in patients with multiple Aspergillomas as well as HIES. Pulmonary angiography and transcatheter embolization should be considered in patients with unresolving hemoptysis as a safe and effective means of treating pulmonary artery pseudoaneurysms.

## Abbreviations

ABPA: Allergic bronchopulmonary aspergillosis
HIES: Hyperimmunoglobulin E syndrome
Ig: Immunoglobulin
IV: Intravenous
